# Complete mitochondrial genome of *Pseudogastromyzon fasciatus* (Osteichthyes: Gastromyzontidae)

**DOI:** 10.1080/23802359.2016.1219641

**Published:** 2016-09-01

**Authors:** Xiaojing Song, Yang Yang, Wenqiao Tang

**Affiliations:** a Laboratory of Fishes, Shanghai Ocean University, Shanghai, China;; b Shanghai Key Laboratory of Marine Animal Taxonomy and Evolution, Shanghai, China

**Keywords:** *Pseudogastromyzon fasciatus*, mitochondrial genome, phylogenetic analysis, Gastromyzontidae

## Abstract

*Pseudogastromyzon fasciatus* belonging to the family Gastromyzontidae is a good model for phylogeny and zoogeography research. The complete mitochondrial genome of *P. fasciatus* was sequenced in this study. The genome sequence is 16,563 bp in length, comprising 13 protein-coding genes, 22 tRNA genes, 2 rRNA genes, and one control region. Overall base composition is 29.70% A, 25.16% T, 16.53% G, and 28.60% C. The result of phylogenetic analysis indicates that *P. fasciatus* mitogenome is close to that of *P. myersi*.

The Gastromyzontidae family comprises 18 genera and approximately 125 species, which inhabit mountain streams in China and Southeast Asia (Nelson et al. 2016). Pectoral and pelvic fins modified into sucker organs for clinging to objects in fast-flowing streams. *Pseudogastromyzon fasciatus* is endemic to the rivers in the coastal area of Southeast China (Yue & Shan 2000) and a good model for phylogeny and zoogeography research. Mitochondrial DNA is a maternally inherited circular genome that serves important functions in metabolism and population genetics (Boore 1999). However, little is known about the complete mitochondrial genome of *P. fasciatus* in GenBank. In this study, the complete mitochondrial genome of *P. fasciatus* was determined. Fish sample was collected from the Xinjiang River (27°53′34.9″ N, 117°38′38.0 E″), Jiangxi, China. The species is stored at Key Laboratory of Aquacultural Resources and Utilization, Shanghai Ocean University, with accession number SLJXWY151010012. The sample DNA is available upon request.

The complete mitogenome of *P. fasciatus* (GenBank accession number: KX101229) is 16,563 bp in length, with overall base composition of 29.70% A, 25.16% T, 16.53% G, and 28.60% C. The circular genome has 13 protein-coding genes, 22 tRNA genes, 2 rRNA genes, and 1control region (CR or D-loop). Apart from the *ND6* gene and eight tRNA genes (*tRNA-Gln*, *Ala*, *Asn*, *Cys*, *Tyr*, *Ser*, *Glu*, and *Pro*) encoded on the L-strand, most genes are on the H-strand. In 13 protein-coding genes, the shortest one is *ATP8* gene (168 bp) and the longest one is *ND5* gene (1839 bp). Twelve of 13 protein-coding genes start with a common initiation codon ATG, while *COI* utilizes GTG. *ND1*, *COI*, *ATP8*, and *ND5* end with TAA; *ATP6*, *COIII*, and *ND4* with TA (incomplete stop codon); *ND2*, *COII*, *ND3* and *Cytb* with T–– (incomplete stop codon); *ND4L* with TTA; *ND6* with TAG. The 22 tRNA genes have lengths ranging from 66 bp (*tRNA^Cys^*) to 76 bp (*tRNA^Lys^*). The 12S and 16S rRNA genes are 954 bp and 1677 bp, respectively. D-loop is 902 bp in length.

To investigate the phylogenetic relationship among Gastromyzontidae family, we downloaded the mitochondrial genome sequences of nine currently available species of Gastromyzontidae, including *Beaufortia kweichowensis* (KX060617), *B. szechuanensis* (KP716708), *Crossostoma lacustre* (AP010774), *Liniparhomaloptera disparis disparis* (AP013301), *Plesiomyzon_baotingensis* (KF732713), *Pseudogastromyzon myersi* (AP013300), *Sewellia lineolate* (AP011292), *Vanmanenia pingchowensis* (KP005457) and *Yaoshania pachychilus* (KT031050), together with African lungfish *Protopterus annectens* (NC018822) as outgroup species. The phylogenetic trees were constructed using MEGA6 (Tamura et al. 2013) for neighbour-joining, maximum-likelihood and maximum parsimony methods. Tree topology was evaluated by 1000 bootstrap replicates. Different methods give the same tree topology, and the result indicates that *P. fasciatus* mitogenome is close to that of *P. myersi* (Figure 1).

**Figure 1.**
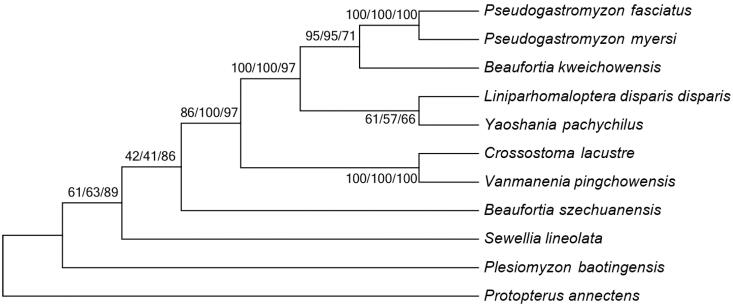
Phylogenetic tree of Gastromyzontidae family, with African lungfish *P. annectens* as an outgroup. The topology of phylogenetic tree was inferred from neighbour-joining, maximum-likelihood and maximum parsimony methods. Bootstrap supports for each analysis are indicated at the nodes.
